# Discrete Serotonin Systems Mediate Memory Enhancement and Escape Latencies after Unpredicted Aversive Experience in *Drosophila* Place Memory

**DOI:** 10.3389/fnsys.2017.00092

**Published:** 2017-12-11

**Authors:** Divya Sitaraman, Elizabeth F. Kramer, Lily Kahsai, Daniela Ostrowski, Troy Zars

**Affiliations:** Division of Biological Sciences, University of Missouri, Columbia, MO, United States

**Keywords:** serotonin, learning, memory, learned helplessness, *Drosophila melanogaster*

## Abstract

Feedback mechanisms in operant learning are critical for animals to increase reward or reduce punishment. However, not all conditions have a behavior that can readily resolve an event. Animals must then try out different behaviors to better their situation through outcome learning. This form of learning allows for novel solutions and with positive experience can lead to unexpected behavioral routines. Learned helplessness, as a type of outcome learning, manifests in part as increases in escape latency in the face of repeated unpredicted shocks. Little is known about the mechanisms of outcome learning. When fruit fly *Drosophila* *melanogaster* are exposed to unpredicted high temperatures in a place learning paradigm, flies both increase escape latencies and have a higher memory when given control of a place/temperature contingency. Here we describe discrete serotonin neuronal circuits that mediate aversive reinforcement, escape latencies, and memory levels after place learning in the presence and absence of unexpected aversive events. The results show that two features of learned helplessness depend on the same modulatory system as aversive reinforcement. Moreover, changes in aversive reinforcement and escape latency depend on local neural circuit modulation, while memory enhancement requires larger modulation of multiple behavioral control circuits.

## Introduction

Skinner coined the term operant conditioning to describe a form of associative learning where organisms learn from the consequences of their own behavior (Skinner, 1950). Mechanisms underlying operant learning have been extensively explored in invertebrate and vertebrate animals as these represent an approach to understand the basis of goal directed behaviors. Operant learning is critically dependent on feedback mechanisms that can modify future decision making and action selection processes. This gives an animal the required flexibility to try out different behaviors in an attempt to better their situation through “outcome learning” (Maier and Watkins, 2005; Heisenberg, 2014, 2015). While operant learning allows for generation of novel solutions and with positive experience can lead to selection of unexpected behavioral routines, the underlying neuronal basis remains largely unexplored.

The external conditions an organism faces can often be unpredictable, uncontrollable and dangerous. As the primary example of outcome learning in learned helplessness, dogs took longer to learn to escape foot shocks after they were exposed to uncontrollable electric shocks (Seligman, 1972). This phenomenon has been investigated in other vertebrate and invertebrate animals, but most intensively in rats and mice (e.g., Maier and Watkins, 2005; Yang et al., 2013; Batsching et al., 2016; Kim et al., 2016). While upregulation of serotonin and corticotrophin-release factor systems within the dorsal raphe nucleus is causally related to development of learned helplessness, other neurochemical systems and brain structures have also been implicated (Maier and Watkins, 2005; Kim et al., 2016).

A key feature of learned helplessness in vertebrate and invertebrate animals is a deficit or delay in escaping/avoiding aversive events, but little is known about the neural mechanisms underlying the increase in escape latency (Maier and Watkins, 2005; Yang et al., 2013; Batsching et al., 2016). When *Drosophila* are exposed to unpredicted aversive temperatures in the heat-box place learning paradigm, flies also increase escape latencies (Wustmann et al., 1996; Sitaraman et al., 2007; Sitaraman and Zars, 2010; Yang et al., 2013; Ostrowski and Zars, 2014; Batsching et al., 2016). Moreover, and intriguingly, flies have a robust place memory when given control of a place/temperature contingency. Serotonin is the only biogenic amine shown to be necessary for *Drosophila* place memory (Sitaraman et al., 2008). It is not clear if the unpredicted exposure induced changes in escape latency and memory require serotonin.

Here we investigated the role of discrete neuronal circuits underlying aversive reinforcement, escape latencies and memory levels in the presence and absence of unexpected aversive events. That is, we asked if serotonin and specific subsets of serotonergic neurons mediate the reinforcing signal for aversive place memory. Furthermore, we explored if specific subsets of serotonergic neurons are necessary and sufficient for the effect of unexpected exposures on increases in escape latency and memory performance. Using an array of genetic tools targeting the serotonin neurons we discovered that aversive reinforcement and escape latency depend on local neural circuit modulation, while memory enhancement requires larger, perhaps bulk, modulation of multiple behavioral control circuits. Thus, two features of learned helplessness, increases in escape latency and changes in memory formation, depend on the same modulatory system as aversive reinforcement. Learned helplessness has been widely cited as a model for anxiety and depression resulting from real or perceived absence of control over the outcome of a situation. In addition to the conserved role of serotonin, our studies in an experimentally tractable system will pave the way for characterizing the precise circuit mechanisms underlying outcome learning.

## Experimental Procedures

### Behavior

Individual flies were conditioned in the heat-box, a set of long narrow chambers. A single fly is allowed to roam in the chamber, and they usually walk from chamber end to chamber end (Zars et al., 2000; Sitaraman et al., 2008). The chamber dimensions are 34 mm long, 1 mm high and 3 mm wide. The top and bottom of the chambers are lined with Peltier elements, and temperature is finely controlled within 0.1°C of a called temperature using custom software and thermocouples (Zars et al., 2000). During training, one half of the chamber is associated with rising temperatures with a pre-determined maximum. That is, when a fly moves to the front half of a chamber, the temperature of the whole chamber rises to a maximum temperature. When the fly goes back across the invisible midline to the rear of the chamber, the whole chamber begins to cool. It takes 3–4 s for the temperature to rise to the maximum or fall again to the cool temperature. Flies typically avoid the chamber-half associated with a high temperature and continue to do so even after the chamber temperature is reset to the preferred 24°C (Wustmann et al., 1996; Kahsai and Zars, 2011). Unexpected exposures were presented as three 1-min exposures to temperatures of 41°C for normal flies (Sitaraman et al., 2007), or other temperatures as indicated for the TrpA1 and TrpM8 experiments. Flies were allowed a rest of 4 min after the unexpected exposure, and then conditioned with a mid-level temperature of 30°C.

Control experiments were done to determine if flies can sense and avoid the temperature used in conditioning. In this case, the temperature in one half of the chamber is raised relative to the control temperature of 24°C. Avoidance of two temperatures, 30 or 41°C, was tested (Zars, 2001). In this case, the rise and fall of temperatures is independent of fly behavior. The response of flies to the temperature gradient is used to test the ability of flies to sense and avoid high temperatures.

The position of each fly is measured every tenth of a second with a spatial resolution of 0.2 mm. Whether a fly is on the punishment associated half of the chamber, or the other side, is used to calculate a Performance Index. The Performance Index is calculated as the time spent on the punishment-associated side minus time spent on the unpunishment associated side divided by total time within a session. There is little ambiguity in where an individual fly is located and when a fly transitions between chamber halves. The maximum error in determining where a fly is located is in the 0.5% range (0.2 mm/34 mm). The maximum error in determining when a fly has transitioned between chamber halves is also small. A fly will typically transition three or four times between chamber halves in a 1 min pre-test phase. This would give a 0.1 s × 4/60 s calculation of about 0.7%. To avoid a side bias in calculations of a Performance Index, approximately 50% of flies are trained to avoid the front half of the chamber. The other 50% are trained to avoid the back half of the chamber. Largely equal numbers of flies from all genotypes were tested in parallel over several weeks. The number of flies from each experiment is listed in the H-statistics in the figure legends. While the behavioral experiments were not done blind to genotype, data is objectively collected with an automated conditioning apparatus and analytic software.

### *Drosophila* Husbandry

Genetic crosses followed typical methods. The GAL4 and effector lines were introgressed with a cantonized white strain (wCS10) and then the X-chromosomes were replaced with a wild-type version in some lines to prevent *white*-mutant effects on learning behavior (Diegelmann et al., 2006). Flies tested for behavior were 2–7 days old, and raised on cornmeal food in an insectary at 25°C, unless otherwise noted, and 60% humidity.

#### Immunohistochemistry

Brains from 4–10 day-old females were dissected in 1× PBS and fixed in 4% paraformaldehyde overnight at 4°C. After 4 × 10-min washing in PAT (0.5% Triton X-100, 0.5% bovine serum albumin in phosphate-buffered saline), tissues were blocked in 3% normal goat serum (NGS) for 90 min, then incubated in primary antibodies diluted in 3% NGS for 12–24 h at 4°C, then washed in PAT, and incubated in secondary antibodies diluted in 3% NGS for 1–2 days at 4°C. Tissues were then washed thoroughly in PAT and mounted using Vectashield (Vector lab, CA, USA) for imaging. Antibodies used were rabbit anti-GFP (Invitrogen A11122) 1:1000, mouse anti-Serotonin (Abcam ab6336) 1:30 and secondary Alexa Fluor 488 and 568 antibodies (1:500). Samples were imaged on a Zeiss 510 confocal microscope (Sitaraman et al., 2008).

#### Generation of Si6 GAL4 and GAL80 Lines

The potential Si6 enhancer was amplified with the primers: GCTTTATTAAATTCCAATTCCCA and TTCGGTTAATTAACTCCTAAGCA. The cloned fragment was subcloned into Gateway donor and the germline transformation vectors pBPGUw with GAL4 and GAL80 regulators (Pfeiffer et al., 2008). Transgenes were targeted to the 3rd chromosome landing site attP2 by the company Genetic Services, Inc. (Sudbury, MA, USA).

### Statistics

Statistical comparisons used non-parametric tests with a Kruskal Wallis ANOVA with multiple comparisons when warranted by significance of the main effect. *P*-values less than 0.05 were considered significant, and marked as *P* < 0.05 = *; *P* < 0.01 = **; *P* < 0.001 = ***; (Kahsai and Zars, 2011). All data were compared with Statistica software version 8.

### Results

#### General Approach

**Figure 1:**
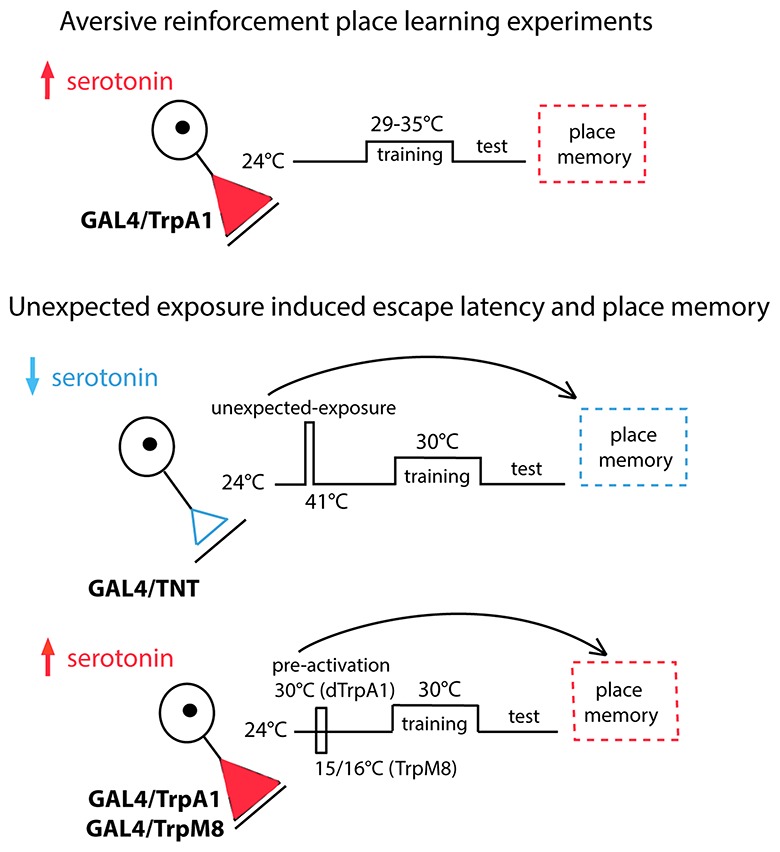
Serotonergic neuron activity was modulated by expressing TrpA1 in specific sets of serotonin neurons (top panel). Place memory levels were tested at 24°C. The necessity of serotonergic neurons was tested by expressing the tetanus toxin light chain (TNT) in these neurons (middle panel). Flies of different genotypes were exposed to 41°C, trained with 30°C and tested at 24°C. The sufficiency of the serotonergic neurons and subsets were examined (lower panel). Serotonin neuron activity was increased by expressing and activating TrpA1 or TrpM8 in specific neurons. Flies were trained at 30°C and tested at 24°C.

We investigated the role of serotonergic neurons and serotonergic neuron subsets in regulating place memory and the effects of unexpected exposures to high temperature on escape latencies and memory levels (Figure 1). In the first set of behavioral experiments, we increased serotonergic neuron activity by expressing TrpA1 in specific sets of serotonin neurons and trained flies at temperatures that activate TrpA1, but are not otherwise reinforcing (Figure 1, top panel). The place memory levels were tested at the baseline temperature of 24°C. In the second set of behavioral experiments, the necessity of serotonergic neurons for changes in escape latencies during training and place memory enhancement after unexpected exposure to high temperature was tested (Figure 1, middle panel). The serotonergic neuron activity was blocked by expressing the tetanus toxin light chain (TNT) in these neurons. Flies of different genotypes were exposed to 41°C and trained with the moderate temperature of 30°C. Place memory was tested at 24°C. In the third set of behavioral experiments, the sufficiency of the serotonergic neurons and subsets were examined for changes in escape latencies and place memory (Figure 1, lower panel). Serotonin neuron activity was increased by expressing TrpA1 or TrpM8 in specific neurons and exposing flies to temperatures that activate these channels. Flies were then trained at 30°C. Escape latencies during training and memory at 24°C was tested.

#### Serotonergic Neurons Are Necessary and Sufficient for Aversive Reinforcement of Place Memory

**Figure 2:**
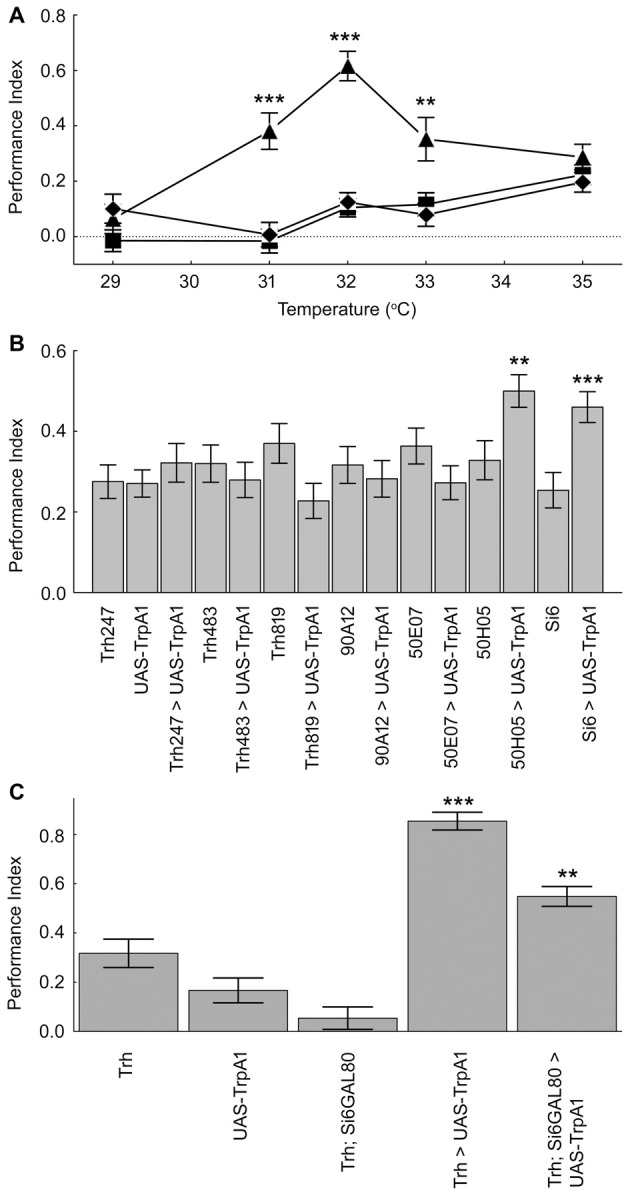
Subsets of the serotonergic system are necessary and sufficient for operant feedback in aversive place memory. **(A)** Activation of serotonergic neurons substitutes for high temperature feedback. Trh-Gal4/TrpA1 (▲), Gal4/+ (⧫) and TrpA1/+ (■) flies were conditioned using 29–35°C. Experimental flies had higher place memories than control flies at three temperatures (29°C *H*_(2, *N* = 274)_ = 5.14, *p* = 0.08; 31°C *H*_(2, *N* = 273)_ = 27.22, *p* < 0.0001; 32°C *H*_(2, *N* = 419)_ = 70.25, *p* < 0.0001; 33°C *H*_(2, *N* = 234)_ = 12.84, *p* = 0.0016; 35°C *H*_(2, *N* = 574)_ = 2.64, *p* = 0.27). **(B)** Flies expressing TrpA1 in subsets of serotonergic neurons with the 50H05 and Si6-GAL4 driver had higher memory scores when trained with 32°C compared to genetic control flies (*H*_(14, *N* = 3627)_ = 56.3, *p* < 0.00001; *P*’s < 0.01 = ** and 0.001 = *** compared to genetic controls after multiple comparisons). **(C)** A subsystem of serotonergic neurons is necessary for normal place memory. TrH-GAL4; TrpA1 flies showed high place memory when conditioned with 32°C compared to genetic control flies, and Si6-GAL80 reduces the induced place memory (*H*_(4, *N* = 1230)_ = 152.7, *p* < 0.0001; *P* < 0.0001 = *** for the Trh-GAL4; TrpA1 compared to control genotypes; *P* < 0.01 = ** for TrH-GAL4/Si6-GAL80; TrpA1 compared to Trh-GAL4; TrpA1 after multiple comparisons). Values represent mean and SEMs in all figures.

The serotonergic system is the only biogenic amine system known to be necessary for *Drosophila* place memory (Sitaraman et al., 2008; Kahsai and Zars, 2011; Ostrowski and Zars, 2014). In the *Drosophila* brain the serotonergic system is comprised of ~40 neurons per hemisphere (Sitaraman et al., 2008; Alekseyenko et al., 2010, 2014; Lee et al., 2011), and these neurons broadly innervate the central brain. To manipulate all or nearly all of these neurons, a Trh-GAL4 driver (Sadaf et al., 2012) was used to drive expression of the thermogenetic effector TrpA1, which can increase neuronal activity at specific temperatures with high temporal precision (Hamada et al., 2008). To address the sufficiency of the serotonergic system in providing aversive reinforcement, extrinsic activation of serotonergic neurons was paired with a behavioral routine. That is, the behavior that takes a fly to one end of the chamber was paired with activation of the serotonergic neurons, thus experimentally closing the loop between behavior and activation of this set of neurons. In this case the aversive high temperature feedback was replaced by temperatures that activate TrpA1 in serotonergic neurons. The temperature range of TrpA1 activation (Pulver et al., 2009) is much lower than those used for high temperature reinforcement allowing for a clear dissociation of serotonin activation and aversive reinforcement. The Trh-GAL4/TrpA1 flies conditioned with 31, 32 and 33°C, temperatures that induce TrpA1 activation, had high memory levels compared to control flies (Figure 2A). Temperatures outside of the activation range of TrpA1 did not support memory formation (Figure 2A). Thus, serotonergic activation can act as an aversive reinforcer (Figure 2).

We next asked if a subset of the serotonergic system can be sufficient for aversive reinforcement in place memory. Seven GAL4 drivers that are expressed in subsets of the serotonergic system were screened for effects on place memory using the TrpA1 effector (Pfeiffer et al., 2008; Lee et al., 2011). These GAL4 lines are from cloned enhancers from genes that are expressed in serotonin neurons (Pfeiffer et al., 2008; Lee et al., 2011), and represent both broad and more restricted expression in the serotonergic neuron set. We had no prediction of which of these lines might influence place memory, but reasoned that this set of GAL4 drivers might identify subsets of critical serotonin neurons since the drivers were expressed in many different serotonin neurons. Of these lines, two were found to have an effect on this direct conditioning. We found that an enhancer from the sixth intron of the SerT gene (Si6-GAL4) when combined with the TrpA1 transgene was sufficient for aversive reinforcement (Figure 2B). When TrpA1 was expressed in the neurons from Si6-GAL4 and flies were trained with 32°C, place memory after training was significantly higher in the experimental flies compared to flies from the control genotypes (Figure 2B). Moreover, a second driver 50H05 when combined with TrpA1 also had significant place memory when conditioned with 32°C.

Flies from all genotypes were tested in control experiments for the ability to sense and avoid a high temperature source. In contrast to conditioning experiments, where temperatures rise and fall depending on where a fly moves in the chamber, the thermosensitivity assay employs a temperature step gradient that is maintained regardless of the behavior of a fly (Zars, 2001). This simpler test asks whether a fly can sense a temperature difference between the preferred 24 and 30 or 41°C. The side of the chamber with the higher temperature was switched when the temperatures changed to force flies to show a temperature preference. Flies from the experimental and control genotypes did not have altered control behaviors (Table 1). Thus, since flies of all genotypes showed that they can sense and avoid high temperatures in the thermosensitivity test, but the experimental flies show an altered memory phenotype indicates that it is memory formation that is specifically altered in these flies.

**Table 1:** Control avoidance behavior in serotonin altered flies.

Genotype	*N*	30°C	41°C
Trh-GAL4/TrpA1	93	0.14 ± 0.05	0.66 ± 0.05
Trh-GAL4/+	93	0.20 ± 0.05	0.72 ± 0.03
TrpA1/+	99	0.16 ± 0.05	0.71 ± 0.04
Ddc-GAL4, THGAL80/TNT	74	0.13 ± 0.08	0.71 ± 0.05
Ddc-GAL4, THGAL80/+	70	0.14 ± 0.08	0.71 ± 0.03
TNT/+	68	0.16 ± 0.06	0.70 ± 0.06
Trh-GAL4/TNT	81	0.11 ± 0.08	0.54 ± 0.06
Trh-GAL4/+	87	0.14 ± 0.07	0.60 ± 0.06
TNT/+	81	0.19 ± 0.07	0.58 ± 0.06
Trh-GAL4/TrpM8	125	0.16 ± 0.07	0.68 ± 0.06
Trh-GAL4/+	129	0.20 ± 0.06	0.73 ± 0.05
TrpM8/+	133	0.19 ± 0.05	0.65 ± 0.05
TrpA1/+	139	0.21 ± 0.02	0.75 ± 0.02
Trh-GAL4/+	110	0.19 ± 0.04	0.75 ± 0.02
Trh-GAL4/TrpA1	106	0.19 ± 0.04	0.73 ± 0.04
Si6-GAL4/+	100	0.27 ± 0.04	0.80 ± 0.03
Si6-GAL4/TrpA1	93	0.21 ± 0.03	0.70 ± 0.03
Trh-GAL4/Si6-GAL80	140	0.22 ± 0.02	0.76 ± 0.02
Trh-GAL4/Si6GAL80/TrpA1	147	0.18 ± 0.03	0.72 ± 0.03

*Statistics: Trh-GAL4/TrpA1: 30°C, *H*_(2, *N* = 285)_ = 1.9, *p* = 0.37; 41°*C* = 0.28, *p* = 0.87. Ddc-GAL4, TH-GAL80/TNT: 30°C, *H*_(2, *N* = 208)_ = 0.66, *p* = 0.72, 41°*C* = 2.43, *p* = 0.29. Trh-GAL4/TNT: 30°C, *H*_(2, *N* = 249)_ = 0.81, *p* = 0.6, 41°*C* = 2.75, *p* = 0.25. Trh-GAL4/TrpM8: 30°C, *H*_(2, *N* = 387)_ = 0.107, *p* = 0.95, 41°*C* = 3.99, *p* = 0.14. Si6-GAL4/TrpA1: 30°C, *H*_(8, *N* = 835)_ = 63.6, *p* < 0.01, *P* < 0.05 for Si6-GAL4/+ compared to TrpA1/+, other relevant P’s = n.s.; 41°*C* = 16.6, *p* < 0.05, P’s n.s. after multiple comparisons*.

The Si6-GAL4 neurons are also necessary for normal place memory. We made an Si6-GAL80 line to suppress the potential activity of GAL4 in these neurons. Si6-GAL80 expresses the GAL80 transcription repressor (GAL4 inhibitor) under the control of Si6 enhancer and thereby restricts/eliminates transgene expression in Si6 positive neurons (Lee et al., 2000). Combining the Si6-GAL80 element with TrH-GAL4 and the TrpA1 transgene led to a partial but significant reduction in the place memory that is formed with activation of all of the serotonergic system using the TrH-GAL4 driver (Figure 2C). Again, flies from the different genotypes did not have altered temperature avoidance (Table 1).

**Figure 3:**
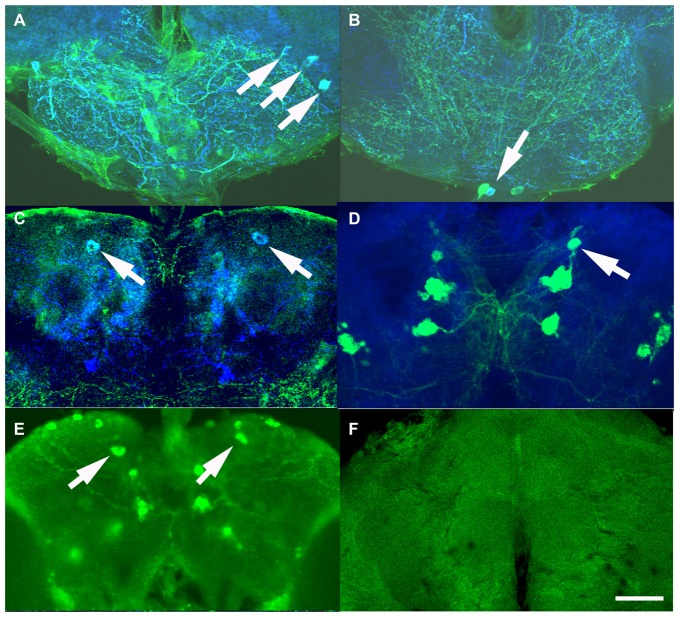
Expression pattern of the serotonin drivers Si6-GAL4, 50H05-GAL4 and Si6-GAL80. **(A–C)** Si6-GAL4 driving UAS-GFP fly brains were co-labeled with anti-GFP (green) and anti-serotonin (blue). **(A)** In an anterior ventral region, three serotonergic neurons are co-labeled (white arrows). A few other small GFP-positive but serotonin negative neurons can also be seen in this region. These SE neurons appear to densely innervate the sub-esophageal ganglion here. **(B)** Multiple GFP neurons are again labeled in this ventral but less anterior section, only one neuron appears to be co-labeled with anti-serotonin (arrow). This serotonergic neuron appears to also innervate the sub-esophageal ganglion. **(C)** In a dorsal posterior section, one pair of large PMP neurons is co-labeled with GFP and anti-serotonin (arrows), termed dorsal PMP neurons (dPMP). **(D)** 50H05-GAL4 driving UAS-GFP brains were co-labeled with anti-GFP (green) and the synapse marker *bruchpilot* (blue). Multiple neurons are labeled in the PMP cluster, including the dPMP neurons. **(E)** Labeling with anti-serotonin in wild-type flies shows multiple PMP neurons, including the dPMP neurons (arrows). **(F)** Addition of a Si6-GAL80 element to the Si6-GAL4 driver suppresses UAS-GFP expression. This is an anterior frontal optical section. Scale bar represents 20 μm in **(A,B)** and 50 μm in **(C–E)**.

We next examined the expression pattern of the Si6- and 50H05 GAL4 drivers. Double labeling experiments (GFP and anti-serotonin) show that Si6-GAL4 drives expression in five serotonergic neurons per brain hemisphere. These include neurons in the SE2 and SE3 clusters and one pair of neuron in the PMP cluster (Figures 3A–C). Based on cell body location, this pair of serotonergic neurons was also identified with the 50H05 driver (Figures 3D,E). 50H05-GAL4 is derived from an intron of the fly serotonin transporter gene and co-labeling with anti-serotonin antibodies revealed that 50H05-GAL4 expresses in 25 serotonergic neurons in each brain hemisphere (Albin et al., 2015). We refer to the neurons that show overlap between the 50H05 and Si6-Gal4 as dorsal (d) PMP neurons, and are different from the DP neurons of Giang et al. (2011) since the dPMP neuron pair is far posterior to the DP neurons. The Si6- and 50H05- GAL4 serotonergic neurons’ innervation pattern includes the sub-esophageal ganglion, the median bundle and discrete parts of the superior medial protocerebrum. When Si6-GAL4 was crossed to Si6-GAL80, all GFP expression in Si6-GAL4-positive neurons was blocked (Figure 3F).

It could be that it is the impact of different numbers of serotonin neurons that influences place memory. The two lines that do influence place memory, Si6-GAL4 and 50H05, label 5 and 25 serotonin neurons per hemisphere, respectively (Albin et al., 2015). The other lines, Trh247-, 483- and 819-GAL4 drivers express in about 15, 12 and 17 serotonin neurons per hemisphere (Lee et al., 2011). The 50E07 drives expression in about 19 serotonin neurons per hemisphere (Jenett et al., 2012). It is not clear how many serotonin neurons are affected by the 90A12 driver since our attempts at labeling detected very weak expression (not shown). Thus, there is not a clear relationship between serotonin cell number and effect on place memory. Taken together, these data suggest that specific subsets of serotonin neurons and their innervation sites are necessary and sufficient for aversive reinforcement in place memory.

#### Serotonergic Neurons Are Necessary and Sufficient for Unexpected High Temperature Exposure Effects on Conditioned Behavior

We next explored if serotonin is also important for outcome learning in the heat-box. After unexpected high temperature exposure wild-type flies increase both escape latencies and memory levels (Sitaraman et al., 2007; Sitaraman and Zars, 2010; Yang et al., 2013; Batsching et al., 2016). Synaptic output from most or all of the serotonergic neurons was blocked using both a DdcGal4;Th-GAL80 driver combination and the Trh-GAL4 driver with the tetanus toxin light chain (TNT; Scholz et al., 2000). Escape latencies were measured as the time it took for individual flies to escape from the punishment-associated half of the chamber during the training session. Consistent with the idea of learned helplessness, this phase was chosen for measurement as it best measures escape from unfavorable conditions after uncontrollable high temperature exposure.

**Figure 4:**
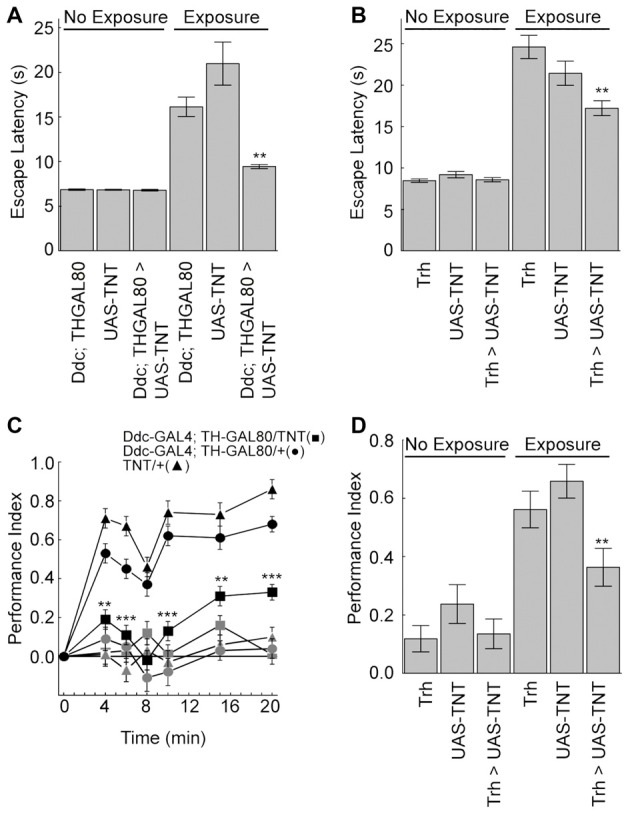
The serotonergic neurons are necessary for the unexpected exposure increases in escape latency and enhancement of place memory. **(A,B)** Unexpected exposure to high temperatures increases escape latencies in control flies, but blocking synaptic transmission with UAS-TNT expression in the serotonergic neurons decreases this effect (Ddc-GAL4; TH-GAL80 experiments (*H*_(5, *N* = 36314)_ = 200.7, *p* < 0.0001; *P* < 0.0001 for exposed control genotypes compared to non-exposed; *P* < 0.01 = ** for exposed experimental flies compared to exposed control genotypes; Trh-GAL experiments (*H*_(5, *N* = 21366)_ = 154.2, *p* < 0.0001; *P* < 0.0001 for exposed control genotypes compared to non-exposed; *P* < 0.01 = ** for exposed experimental flies compared to exposed control genotypes). **(C)** Flies with the tetanus toxin light chain (UAS-TNT) expressed with Ddc-GAL4; TH-GAL80 serotonergic neurons (■) and control genotypes (•) and (▲) were conditioned for 4–20 min at 30°C with (dark symbols) or without (light symbols) unexpected exposure. Expression of TNT in the serotonergic neurons reduces the enhancement of place memory (4 min, *H*_(5, *N* = 553)_ = 132.25, *p* < 0.0001; 6 min, *H*_(5, *N* = 486)_ = 124.05, *p* < 0.0001; 8 min, *H*_(5, *N* = 428)_ = 57.85, *p* < 0.0001; 10 min, *H*_(5, *N* = 433)_ = 125.41, *p* < 0.0001, 15 min, *H*_(5, *N* = 477)_ = 103.74, *p* < 0.0001; 20 min, *H*_(5, *N* = 615)_ = 212.66, *p* < 0.0001; *P*’s < 0.01 = ** and < 0.001 = *** comparing the experimental group to control genotypes after multiple comparisons). **(D)** Unexpected enhancement of place memory requires Trh-GAL4 neurons (*H*_(5, *N* = 734)_ = 97.6, *p* < 0.0001; *P* < 0.01 = ** comparing the experimental group to control genotypes after multiple comparisons).

Flies from the genetic control genotypes strongly increased the escape latency with exposure to high temperatures (Figures 4A,B). Flies with blocked serotonergic synaptic transmission had a significant reduction in the escape latency when exposed to unexpected high temperatures compared to normal flies (Figures 4A,B). Moreover, genetic control flies showed the expected increase in memory levels with unexpected high temperature exposure, which was dampened when TNT was expressed in the serotonergic neurons (Figures 4C,D). Finally, flies from the tested genotypes had no significant changes in control behaviors (Table 1). Thus, the serotonergic system is necessary for the increases in escape latencies and memory performance after unexpected high-temperature exposure.

**Figure 5:**
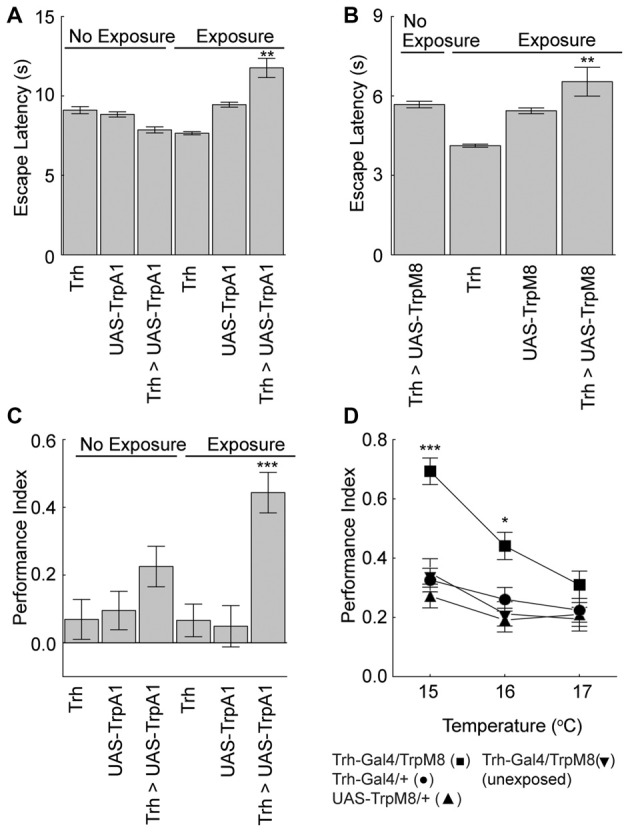
Serotonergic neurons mediate unexpected exposure increases in escape latencies and enhancement of place memory. **(A)** Flies were exposed to 32°C to activate the Trh-GAL4-positive neurons with UAS-TrpA1 expression, which increased escape latencies (*H*_(5, *N* = 35809)_ = 1259.8, *p* < 0.0001; *P* < 0.01 = ** experimental flies with unexpected exposure compared to control genotypes and non-exposed flies after multiple comparisons). **(B)** Flies were exposed to 15°C cool temperatures to activate the Trh-GAL4 neurons with UAS-TrpM8, which increased escape latencies (*H*_(3, *N* = 11681)_ = 384.3, *p* < 0.0001; *P* < 0.01 = ** experimental flies with unexpected exposure compared to control genotypes and non-exposed flies after multiple comparisons). **(C)** Flies expressing the TrpA1 in serotonergic neurons with the Trh-GAL4 driver had an enhanced memory after unexpected exposure to a warm temperature compared to genetic control flies (*H*_(5, *N* = 737)_ = 31.9, *p* < 0.0001; *P* < 0.001 = *** compared to all other groups after multiple comparisons). **(D)** Flies expressing the cool responsive TrpM8 with the Trh-GAL4 driver (Trh-GAL4/TrpM8 (■)) had enhanced memory levels compared to genetic control flies (Trh-GAL4/+ (•) and UAS-TrpM8/+(▲)) and GAL4/TrpM8 flies not exposed to a low temperature (▼) (15°C, *H*_(3, *N* = 516)_ = 48.1, *p* < 0.0001; 16°C, *H*_(3, *N* = 536)_ = 17.5, *p* = 0.0006; 17°C, *H*_(3, *N* = 514)_ = 3.6, *p* = 0.3; *P*’s < 0.001 = *** and < 0.05 = * of the experimental genotype with exposure compared to all other groups with multiple comparisons).

We next asked if the serotonergic neurons were also sufficient for the unexpected exposure effects on escape latencies and memory. Flies expressing TrpA1 or the cool responsive TrpM8 (Peabody et al., 2009) in the serotonergic neurons were exposed to activating temperatures. Activation of serotoninergic neurons with either effector led to an increase in the escape latency compared to genetic and no-exposure control groups (Figures 5A,B). Moreover, activation of serotonergic neurons with either effector under maximal conditions nearly doubled place memory compared to genetic and conditioning control flies (Figures 5C,D). Flies from the different genotypes did not have altered control behaviors (Table 1). Thus, extrinsic activation of serotonergic neurons can induce unexpected exposure changes in escape latency and place memory.

**Figure 6:**
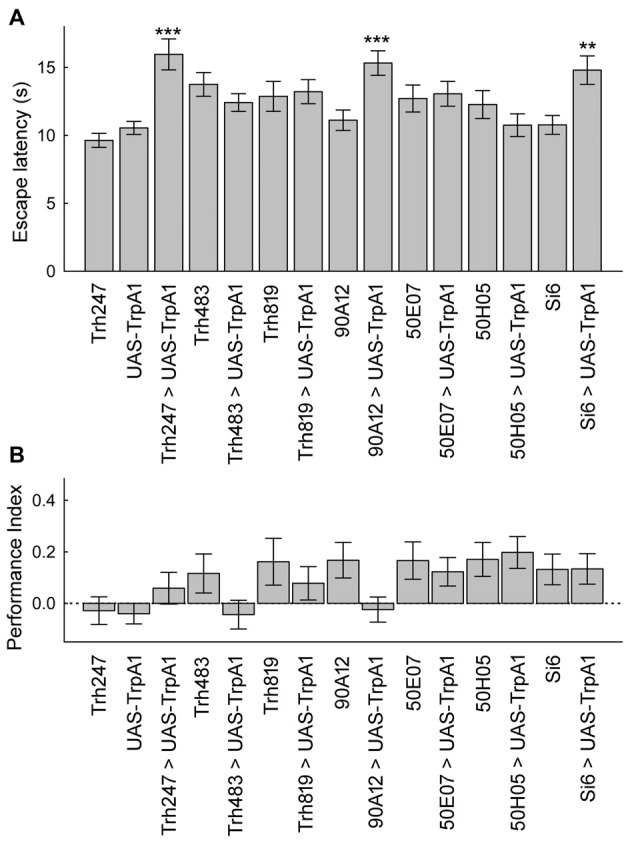
A subset of serotonin drivers can enhance escape latencies, but none can enhance memory with unexpected exposures. **(A)** Flies from three genotypes expressing UAS-TrpA1 had increases in escape latencies after unexpected exposures (*H*_(14, *N* = 17027)_ = 355.1, *p* < 0.0001; *P*’s < 0.01 = ** and < 0.001 = *** compared to control genotypes). **(B)** Memory was not altered in the different genotypes after unexpected exposures (*H*_(14, *N* = 1829)_ = 33.0, *p* = 0.003; *P*’s = n.s. between all groups after multiple comparisons).

Finally, we explored whether or not subsets of serotonergic neurons could alter escape latencies or memory levels with pre-training activation. We tested seven GAL4 lines because of the broad and more restricted expression in the serotonin neuron set. These are the same GAL4 drivers that were previously examined for direct conditioning of place memory. Activation of neurons with three serotonin GAL4 drivers in a pre-test phase significantly increased escape latency (Figure 6A). By contrast, none of the drivers altered place memory after pre-training activation of these neurons (Figure 6B). Thus, while a subset of the serotonin neurons can alter escape latencies upon activation, only activation of large portions of the serotonergic system can induce the memory enhancing effect.

### Discussion

Operant learning, where an animal selects one of several potential behaviors to increase reward or reduce punishment plays a key role in development of goal-directed behaviors. Central to this form of learning is feedback that helps an animal select an appropriate behavior. In our previous work we showed that flies quickly learn to avoid spatial positions associated with aversive high temperature and this avoidance is disrupted by manipulation of the serotonin system (Sitaraman et al., 2008). Although other biogenic amines like dopamine and octopamine play critical roles in other forms of learning in *Drosophila*, they do not influence operant place memory (Sitaraman et al., 2008, 2010). What was unclear was the function of the serotonin neurons in other aspects of operant place learning and memory.

We substituted high temperature punishment in place learning with activation of serotonin neurons and discovered that serotonin release mediates aversive reinforcement in place memory. Furthermore, we find that specific subsets of serotonin neurons labeled by 50H05-GAL4 (expressed in 25 serotonin neurons) and Si6-GAL4 (expressed in 5 serotonin neurons) are sufficient in mediating aversive reinforcement. Since the 5 serotonin neurons labeled by Si6-GAL4 are also found in 50H05 we concluded that these neurons are critical in signaling aversive reinforcement. These neurons innervate the fly brain in several regions, including the sub-esophageal ganglion, the median bundle, and superior medial protocerebrum. This innervation pattern suggests that these neurons can influence multiple neural sites. A deeper investigation of neurons within these regions that express serotonin receptors will illuminate the circuit pathways by which serotonin mediates aversive reinforcement.

Using the same operant learning paradigm we discovered a novel pre-exposure phenomenon where pre-exposure to unpredicted high temperature enhances place memory formation. Interestingly, the memory enhancement is only observed when conditioning uses low temperature reinforcement of 27–30°C (Sitaraman et al., 2007; Sitaraman and Zars, 2010). Based on these experiments we hypothesized that unpredictable exposures induce a state change in the nervous system that somehow stores the unpredicted high temperature exposure effects until a predictable read-out is determined. When released, this stored information then promotes higher than typical memory levels. The neural identity that stores the unexpected exposure effect was unknown. We asked if the serotonin system mediates the effects of unexpected exposure on memory performance.

Broad manipulation of serotonin system shows that induced serotonin release substitutes the unpredictable high temperature exposure and phenocopies the increase in memory performance. Our analysis of subsets of serotonin neurons reveals that it is only with a large portion of the serotonergic system that a place memory enhancement through unexpected activation can be induced.

Unpredicted aversive events, including high temperature, electric shock and vibration have profound effects on escape latencies and motivated climbing in *Drosophila* (Yang et al., 2013; Batsching et al., 2016; Ries et al., 2017). Results from our experiments confirm that high temperature exposure increases escape latencies. We discovered that the same small set of serotonin neurons that mediate conditioning also mediate the increase in escape latency with exposure to high temperatures. This result is generally in line with the un-signaled vibration induced hesitation of climbing behavior as also requiring the serotonin neurons. Whether or not the same specific set of serotonin neurons are critical for electric shock induced changes in escape latencies awaits future studies (Batsching et al., 2016). It is likely that the vibration induced changes in motivated climbing requires a different set of serotonin neurons since Ries et al. (2017) focus on serotonin neurons that innervate the mushroom bodies. The mushroom bodies are not required for place memory (Wolf et al., 1998).

Animal models of depression and anxiety have been studied intensively for decades as they might help unravel the mechanistic basis of these conditions and aid development of pharmacological and therapeutic approaches (Abelaira et al., 2013; Logan and McClung, 2016). In most animal models, lack of motivation to perform key behaviors as a result of internal and external stressors has been widely studied in relation to depression. In the first animal model of learned helplessness, dogs lost the motivation to escape/avoid punishment following exposure to unpredictable, uncontrollable electric shocks (Seligman and Maier, 1967). Continued study of this condition in rodents has illuminated several genetic, molecular, and cellular targets (Maier and Watkins, 2005). Drugs targeting serotonin are regularly prescribed to alleviate symptoms associated with stress, anxiety, and depression in human patients. Continued studies from animal models will hopefully point to other drug targets (Hamon and Blier, 2013). Our results highlight the role of serotonin as a modulator of two features of learned helplessness, and provides a promising model to understand neurobiological basis of depression and anxiety.

## Author Contributions

TZ, DS, EFK, LK and DO: designed approach and experiments; collected data and performed data analysis. DS, EFK, LK and DO: performed experiments. DS and TZ: wrote article with contributions from all authors.

## Conflict of Interest Statement

The authors declare that the research was conducted in the absence of any commercial or financial relationships that could be construed as a potential conflict of interest. The reviewer MS and handling Editor declared their shared affiliation, and the handling Editor states that the process nevertheless met the standards of a fair and objective review.
